# CT radiomics for noninvasively predicting NQO1 expression levels in hepatocellular carcinoma

**DOI:** 10.1371/journal.pone.0290900

**Published:** 2023-09-11

**Authors:** Zenglei He, Xiaoyong Shen, Bin Wang, Li Xu, Qi Ling

**Affiliations:** 1 Department of Hepatobiliary and Pancreatic Surgery, The First Affiliated Hospital, Zhejiang University School of Medicine, Hangzhou, Zhejiang, PR China; 2 Department of Radiology, The First Affiliated Hospital, Zhejiang University School of Medicine, Hangzhou, Zhejiang, PR China; Affiliated Hospital of Nanjing University of Chinese Medicine: Jiangsu Province Academy of Traditional Chinese Medicine, CHINA

## Abstract

Using noninvasive radiomics to predict pathological biomarkers is an innovative work worthy of exploration. This retrospective cohort study aimed to analyze the correlation between NAD(P)H quinone oxidoreductase 1 (NQO1) expression levels and the prognosis of patients with hepatocellular carcinoma (HCC) and to construct radiomic models to predict the expression levels of NQO1 prior to surgery. Data of patients with HCC from The Cancer Genome Atlas (TCGA) and the corresponding arterial phase-enhanced CT images from The Cancer Imaging Archive were obtained for prognosis analysis, radiomic feature extraction, and model development. In total, 286 patients with HCC from TCGA were included. According to the cut-off value calculated using R, patients were divided into high-expression (n = 143) and low-expression groups (n = 143). Kaplan–Meier survival analysis showed that higher NQO1 expression levels were significantly associated with worse prognosis in patients with HCC (*p* = 0.017). Further multivariate analysis confirmed that high NQO1 expression was an independent risk factor for poor prognosis (HR = 1.761, 95% CI: 1.136−2.73, *p* = 0.011). Based on the arterial phase-enhanced CT images, six radiomic features were extracted, and a new bi-regional radiomics model was established, which could noninvasively predict higher NQO1 expression with good performance. The area under the curve (AUC) was 0.9079 (95% CI 0.8127–1.0000). The accuracy, sensitivity, and specificity were 0.86, 0.88, and 0.84, respectively, with a threshold value of 0.404. The data verification of our center showed that this model has good predictive efficiency, with an AUC of 0.8791 (95% CI 0.6979–1.0000). In conclusion, there existed a significant correlation between the CT image features and the expression level of NQO1, which could indirectly reflect the prognosis of patients with HCC. The predictive model based on arterial phase CT imaging features has good stability and diagnostic efficiency and is a potential means of identifying the expression level of NQO1 in HCC tissues before surgery.

## Introduction

Liver cancer is the sixth most common malignancy, making it the third leading cause of cancer-related mortality worldwide. Hepatocellular carcinoma (HCC) accounts for 75–85% of the total liver cancer burden [1–3]. Although surgical resection and liver transplantation are considered effective for HCC treatment, postoperative recurrence remains common and may result in poor outcomes. Classical prognostic indicators, including pathological features, laboratory diagnostic indicators such as alpha-fetoprotein (AFP), and other examination methods, including CT, MRI, and ultrasound, fail to meet the clinical needs of precision and personalized medical strategies. Additional exploration of new prognostic markers is needed to stratify the prognosis of patients and contribute to the development of more effective individualized treatment strategies.

The NAD(P)H quinone oxidoreductase 1 gene (*NQO1*), which is located on chromosome 16q22.1, encodes reductive coenzyme/quinone oxidoreductase, also known as DT-nicotinamide dehydrogenase, which is a homodimeric flavoprotein that catalyzes the two-electron reduction of quinone to hydroquinone. Together, NQO1 and other phase I and phase II metabolic enzymes constitute the metabolic network of exogenous toxic substances *in vivo*, playing an important role in the detoxification metabolism [4]. NQO1 is widely distributed in human organs, with the highest expression levels observed in the gastrointestinal tract, gall bladder, fat tissue, and thyroid [5]. Although NQO1 is not abundantly expressed in the normal liver, its mRNA levels in HCC tissues were found to be approximately 50 times higher than that in normal tissue [6]. High NQO1 expression is associated with tumor metastasis, angiogenesis, and poor prognosis. NQO1 is highly expressed in human and mouse HCC cells, promoting proliferation and mediating tumor growth by activating the PI3K/AKT and MAPK/ERK signaling pathways, which suggests that NQO1 can be used as a therapeutic target for HCC [7]. In an HCC orthotopic transplantation tumor model, both NQO1 knockout and an NQO1 inhibitor blocked tumor growth and induced apoptosis, suggesting that NQO1 plays an important role in maintaining the proliferation of HCC cells [8]. NQO1-responsive prodrugs and nanocarriers have been developed for cancer treatment [9–12]. Notably, the ClinicalTrials.gov website (https://www.clinicaltrials.gov) contains 36 NQO1-relevant clinical trials.

Radiomics provides noninvasive and high-throughput imaging via a technique called "image sequencing" and dynamically detects and quantitatively reflects tumor characteristics. Using automated algorithms, radiomics allows the high-throughput extraction of features from digital medical images in a region of interest. In previous studies, radiomics has been shown to be useful for early diagnosis, typing, tumor heterogeneity and microenvironment evaluation, and preoperative microvascular invasion estimation in HCC [13–15].Certain genetic markers or immunophenotypes in liver cancer tissues are associated with aggressive tumor behavior and poor prognosis [16, 17]. However, techniques for measuring them require invasive tissue sampling and specialized equipment. To the best of our knowledge, no study has used radiomic features to predict NQO1 expression levels in HCC and patient outcomes. Therefore, the present study aimed to investigate the associations between NQO1 expression and the prognosis of patients with HCC, NQO1-related immune infiltration phenotype, and related gene analysis. In addition, radiological models of the whole tumor and peritumoral areas obtained using CT radiomics technology from The Cancer Genome Atlas (TCGA) and The Cancer Imaging Archive (TCIA) were first introduced to evaluate whether the radiomics model could noninvasively predict NQO1 expression levels. Our findings provide valuable insights regarding the noninvasive prediction of tissue biomarkers using radiomics.

## Materials and methods

### Data and image sources

**Fig 1:**
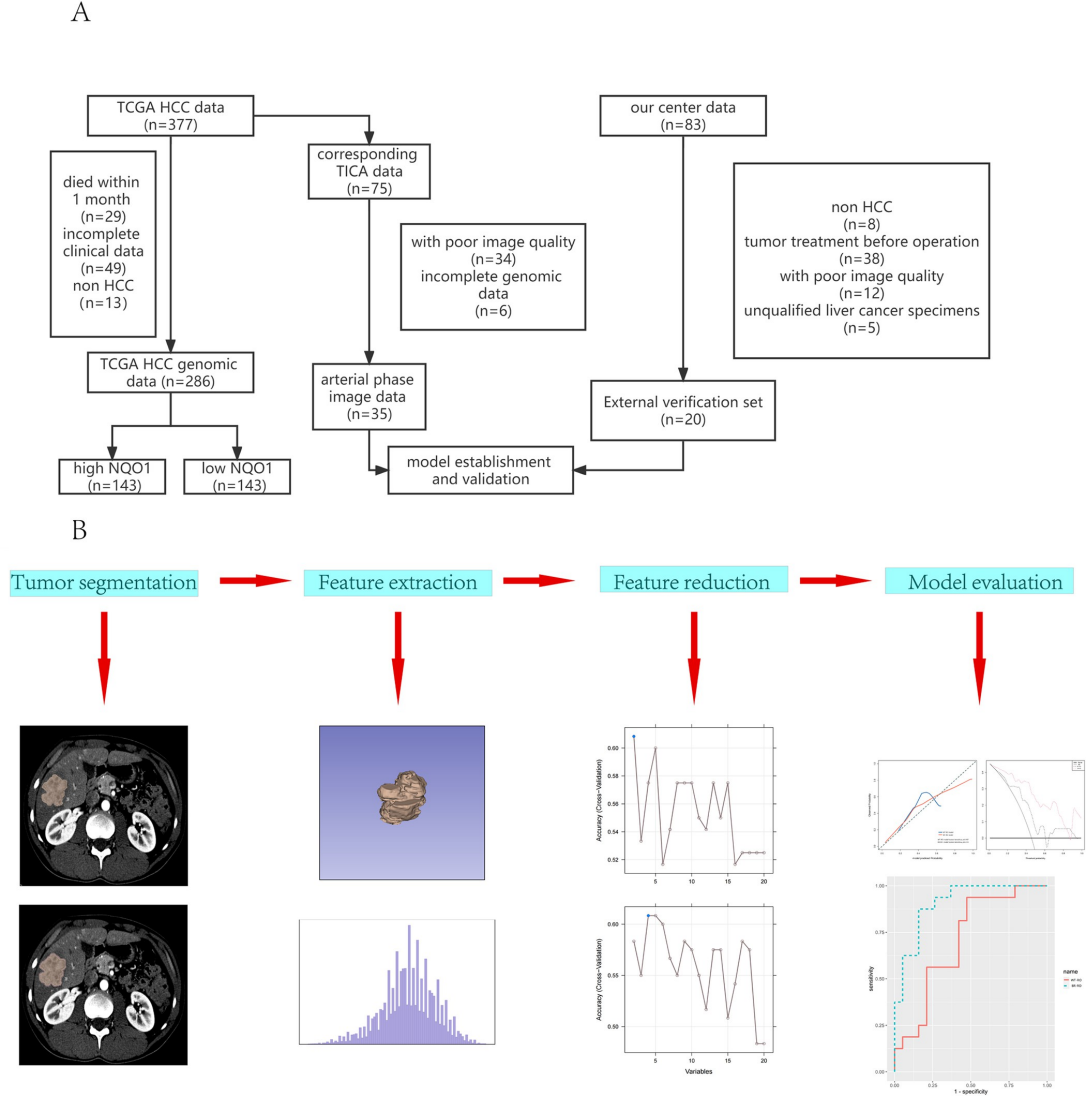
Flowchart of data collection and analysis. (A) Gene and image data screening process. (B) Flowchart of radiomic progression. TCGA: The Cancer Genome Atlas; TCIA: The Cancer Imaging Archive; HCC: hepatocellular carcinoma; NQO1: NAD(P)H: quinone oxidoreductase 1; WT-RO: whole-tumor regional radiomics; BR-RO: bi-regional radiomics.

A total of 377 liver cancer case records were downloaded from TCGA data portal (https://portal.gdc.cancer.gov/). Twenty-nine patients died within 1 month after surgery, 13 patients without HCC and 49 patients with missing data were excluded. Therefore, a total of 286 patients were included in the study. Among them, 75 patients had preoperative CT images stored in TCIA (https://www.cancerimagingarchive.net/). Samples with no arterial phase enhancement images, samples with poor image quality, or images with no corresponding clinical or gene expression information were excluded. A total of 35 cases were considered for radiomic analysis. External validation data came from 83 cases of patients who underwent liver surgery at The First Affiliated Hospital, College of Medicine, Zhejiang University from November 2021 to December 2021, including eight cases of non-liver cancer, 38 cases of preoperative tumor treatment, 12 cases with no artery phase images or poor artery phase image quality preoperative CT, and five cases with unqualified liver cancer specimens. A total of 20 outlier validation data were finally included. Our study protocol was approved by the clinical ethics review board of The First Affiliated Hospital, College of Medicine, Zhejiang University, and written informed consent was obtained from the patients. A brief flowchart of the process is shown in **Fig 1A**. The authors had no access to information that could identify any individual participants during or after data collection.

### Identification of NQO1 as a differentially expressed gene

UCSC XENA (https://xenabrowser.net/datapages/), with the Toil process, was used to unify the handling of TCGA, GTEx, and transcript per million (TPM) RNA-seq data formats [18]. HCC data from TCGA and the corresponding normal tissue data from GTEx were extracted. For data preprocessing, we first transformed the NQO1 expression data into the TPM format. Next, we transformed the TPM values into a log_2_ (TPM+1) scale for downstream analysis by adding 1 to each value of TPM to avoid divergence between zero TPMs. The second step was to merge gene data with corresponding clinical data. Finally, using the merged data, all eligible cases were divided into high- and low-expression groups based on the NQO1 expression cut-off value. Kaplan–Meier analysis was used to assess the overall survival (OS) rates of both groups, and a Kaplan–Meier curve was plotted. The prognostic value of multiple variables, including NQO1 levels, sex, age, tumor grade and stage, AFP levels, and vascular invasion, were evaluated via univariate and multivariate Cox proportional hazard regression analysis.

### Correlation analysis of NQO1 with HCC clinical and pathology features

Univariate Cox regression was used to conduct exploratory subgroup analysis to analyze the effect of different expression levels of NQO1 on the prognosis of patients in different subgroups of each covariable. The Spearman’s rank correlation coefficient was used to analyze the correlation between NQO1 and tumor clinical and pathological features, and the results are shown by a correlation heat map.

### NQO1-related immunoinfiltration phenotype and related gene analysis

The gene expression matrix of HCC samples was uploaded to the CIBERSORTx database (https://cibersortx.stanford.edu/), and the immunoinfiltration of each sample was calculated. The R package “limma” was used to analyze the difference in the degree of immune cell infiltration between the groups. Correlation between NQO1 and immune genes was analyzed using Spearman’s rank correlation coefficients, and a heat map was constructed to visualize the results.

### Image segmentation, feature extraction, and feature selection

The whole-tumor region (i.e., the region of tumor segmentation) was manually outlined using 3D-slicer software (ver. 4.10.2) under the supervision of a hepatobiliary surgeon; more information can be found in previous studies [19, 20]. Using Python’s SimpleITK package (https://simpleitk.org/), the whole-tumor and 3 mm peritumoral regions were obtained by taking the whole-tumor region and automatically ballooning it outward; we termed this the whole-peritumor region. Finally, 107 standard imaging features were included using R (pyradiomics package). The correlation and redundancy between the image omics features result in overfitting of the model, affecting the generalization ability of the prediction model of *NQO1* expression. Recursive feature elimination (RFE) based on the maximum correlation minimum redundancy (mRMR) algorithm was used. First, the importance of the features was ranked by the algorithm, and then the features that contributed the least to the model were eliminated. The iterative process was repeated several times until the number of remaining features reached the required number.

### Model establishment

Logistic regression models were constructed using the best feature subsets from the tumor region or bi-regional area. The area under the curve (AUC) was calculated to evaluate the performance of the radiomic models using receiver operating characteristic curves (ROC). Model predictive accuracy was assessed using calibration curves. As a final step, a decision curve analysis (DCA) was performed to determine the clinical value of radiomic evaluation. A research flowchart of the current study is shown in **Fig 1B**.

### Statistical analysis

R version 4.1.0 (http://www.r-project.org/) (packages including limma, pROC, rms, glmnet, rmda, survminer, survival, mRMRe, irr, and caret) were used for statistical analysis of data. Quantitative variables are expressed as the mean ± SD or median and quartiles. Categorical variables such as sex are presented as values and percentages. Student’s *t*-test or Wilcoxon’s rank-sum test was used to compare quantitative variables. The chi-square test was used to compare categorical variables. The Kaplan–Meier method and log-rank test were used for survival analysis. Multivariate Cox regression analysis was used to determine independent risk factors for OS. We calculated the AUCs of the models and compared them using De Long’s method [21]. Differences were considered statistically significant at a two-sided *p*-value of 0.05. Significance indicators: ns, non-significant (*p*≥0.05); *, *p*<0.05; **, *p*<0.01; and ***, *p*<0.001.

## Results

### Patient characteristics

A total of 286 patients with HCC from TCGA database were included for further analysis. The median follow-up time was 629 days (Quartile: 386–1247 days). The cut-off value of the NQO1 expression level calculated using the R package “survminer” was 2.2177. According to the obtained cut-off value, the patients were divided into a high-expression group (n = 143) and a low-expression group (n = 143) **(Table 1)**. Only vascular invasion showed significant differences between the two groups; other parameters, including age, sex, stage, grade, hepatic inflammation, residual tumor, and AFP levels, showed no significant differences.

**Table 1:** Baseline characteristics of patients with HCC.

Variable	Total (n = 286)	Low (n = 143)	High (n = 143)	*p-*value
Age, n (%)				0.193
~59	138 (48)	63 (44)	75 (52)	
60~	148 (52)	80 (56)	68 (48)	
Gender, n (%)				0.161
Female	90 (31)	51 (36)	39 (27)	
Male	196 (69)	92 (64)	104 (73)	
Grade, n (%)				0.181
G1/G2	176 (62)	94 (66)	82 (57)	
G3/G4	110 (38)	49 (34)	61 (43)	
Stage, n (%)				0.999
I/II	213 (74)	107 (75)	106 (74)	
III/IV	73 (26)	36 (25)	37 (26)	
Hepatic_inflammation, n (%)				0.802
None	97 (34)	51 (36)	46 (32)	
Unknown	92 (32)	44 (31)	48 (34)	
Mild/Severe	97 (34)	48 (34)	49 (34)	
Residual_tumor, n (%)				0.999
R0	264 (92)	132 (92)	132 (92)	
R1/R2/RX	22 (8)	11 (8)	11 (8)	
AFP (ng/ml), n (%)				0.777
~399	153 (53)	79 (55)	74 (52)	
400~	73 (26)	34 (24)	39 (27)	
Unknown	60 (21)	30 (21)	30 (21)	
Vascular_invasion, n (%)				<0.001
None	160 (56)	97 (68)	63 (44)	
Unknown	40 (14)	15 (10)	25 (17)	
Micro/Macro	86 (30)	31 (22)	55 (38)	

Grade: tumor grade; Stage: pathologic stage based on AJCC^7th^; AFP: alpha-fetoprotein

### Higher NQO1 level was associated with worse HCC prognosis

**Fig 2:**
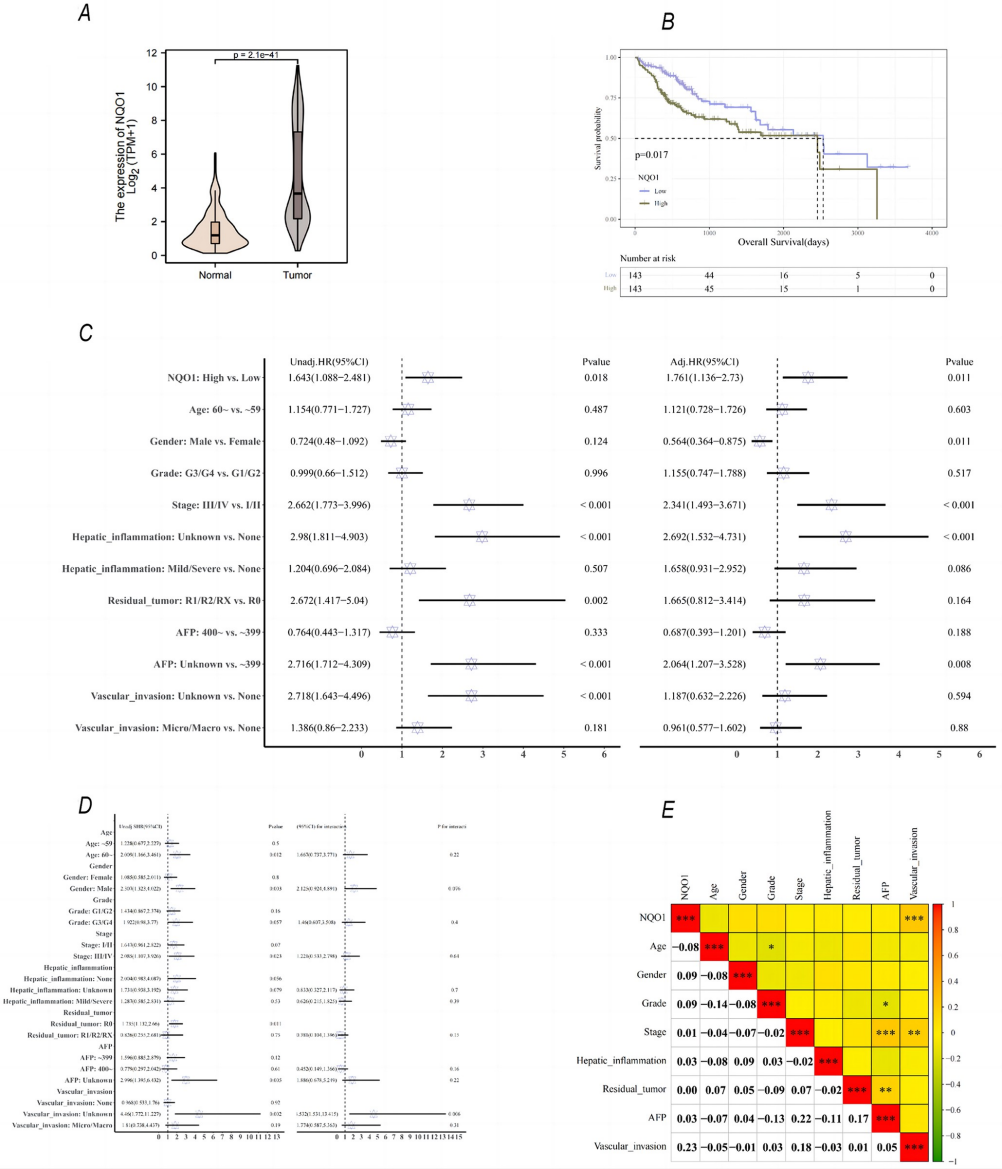
Differential expression of *NQO1* in cancer and para-cancer tissues, its relationship with prognosis and clinical variables, and its effect on subgroups. (A) The expression level of *NQO1* in cancer tissues was higher than that in adjacent tissues, and the median difference between the two groups was 2.261 (1.875–2.891), indicating a significant difference (*p*<0.001). (B) The median survival time of the low-expression group was 2532 days and that of the high-expression group was 2456 days. Kaplan–Meier curve showed that higher NQO1 expression was significantly associated with lower overall survival (*p* = 0.017). (C) High expression of NQO1 is an independent risk factor for postoperative overall survival in patients with HCC (HR = 1.761, 95% CI: 1.136−2.730, *p* = 0.011). (D) Univariate Cox regression was used to conduct exploratory subgroup analysis to analyze the effect of NQO1 (high-expression group vs. low-expression group) on the prognosis of patients in different subgroups of each covariable. (E) Correlation analysis between NQO1 and clinical indicators; heat map shows that the NQO1 was significantly correlated with vascular invasion (*p*<0.001).

Transcriptome sequencing data of NQO1 from TCGA database (https://portal.gdc.cancer.gov/) were analyzed. NQO1 levels were higher in the tumor group than in the normal group (*p*<0.001), with the median difference between the two groups being 2.261 (1.875–2.891) **(Fig 2A)**. Kaplan–Meier survival analysis revealed that high levels of NQO1 were associated with a worse prognosis **(Fig 2B)** (*p* = 0.017). Univariate Cox regression analysis showed that high NQO1 expression level was a risk factor for poor prognosis (hazard ratio [HR] = 1.643, 95% confidence interval [CI]: 1.088−2.481, *p* = 0.018) **(Fig 2C)**. Further multivariate analysis confirmed that high NQO1 expression was an independent risk factor for poor prognosis (HR = 1.761, 95% CI: 1.136−2.73, *p* = 0.011) **(Fig 2C)**.

Univariate Cox regression was used to conduct exploratory subgroup analysis to analyze the effect of NQO1 on the prognosis of patients in different subgroups for each covariable. No significant interaction was observed between the expression level of NQO1 and age, sex, stage, grade, hepatic inflammation, residual tumor, and AFP levels; however, a significant interaction was observed between NQO1 and vascular invasion **(Fig 2D)**. Furthermore, we analyzed the correlation between NQO1 and the clinical features of tumors, and the results of the correlation heat map showed that NQO1 was significantly correlated with vascular invasion (*p*<0.001) **(Fig 2E)**.

### Immune infiltration phenotype and immune gene analysis

**Fig 3:**
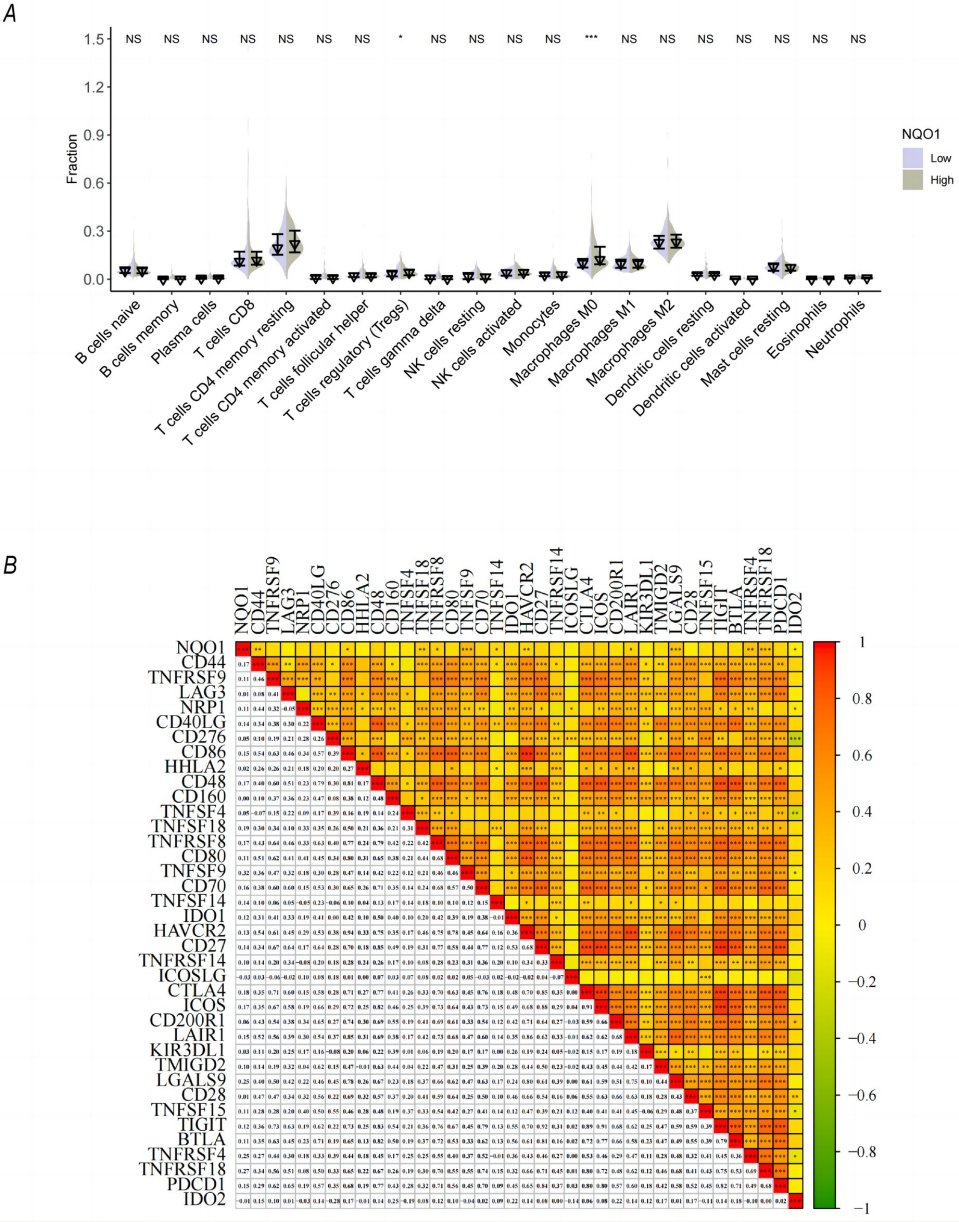
Infiltration of immune cells in HCC and analysis of correlation with immune gene expression. (A) In the high-expression group, the infiltration degree of M0 macrophages was significantly increased (*p*<0.05), whereas the infiltration degree of naïve B cells was not significantly different between the two groups (*p*>0.05). (B) Spearman’s rank correlation coefficient was used to analyze the correlation between NQO1 and immune genes, and it was found that NQO1 was significantly correlated with CD44, TNFSF18, TNFSF9, and other immune genes.

The infiltration of immune cells in HCC was analyzed, and 20 immune cell types are shown in **Fig 3A**. The infiltration degree of M0 macrophages and regulatory T cells was significantly increased in the group with high NQO1 expression levels. However, there was no significant difference in the degree of infiltration of other immune cells, including naïve B cells, memory B cells, NK cells, M1 and M2 macrophages, dendritic cells, and neutrophils, between the two groups. In addition, correlation analysis between NQO1 and immune genes was conducted (**Fig 3B**). NQO1 was significantly correlated with immune genes, such as *CD44*, *CD86*, *TNFSF18*, *TNFRSF8*, *TNFSF9*, *TNFSF14*, *HAVCR2*, *LAIR1*, *LGALS9*, and *IDO2*, among which *TNFSF9* and *LGALS9* had the strongest correlation.

### Construction and validation of radiomic models for predicting NQO1 expression levels

The median (intraclass correlation coefficient [ICC]) value of the imaging features based on the whole-tumor region was 0.979; there were 90 imaging features with an ICC value greater than 0.75 (84.1% of the total features). The median ICC value of the image features based on the whole-peritumor region was 0.971; there were 102 image features with ICC values greater than 0.75 (95.3% of the total features). The ICC values of the selected imaging features were all above 0.8 **(Table 2)**.

**Table 2:** Intraclass correlation coefficient (ICC) values for radiomics features.

Radiomics features	ICC
Whole_glszm_SmallAreaEmphasis	0.977876
Whole_ngtdm_Coarseness	0.938499
Whole_peri_glrlm_RunLengthNonUniformity	0.976227
Whole_peri_ngtdm_Coarseness	0.916978
Whole_peri_firstorder_TotalEnergy	0.975062
Whole_peri_glszm_SmallAreaLowGrayLevelEmphasis	0.841527

**Fig 4:**
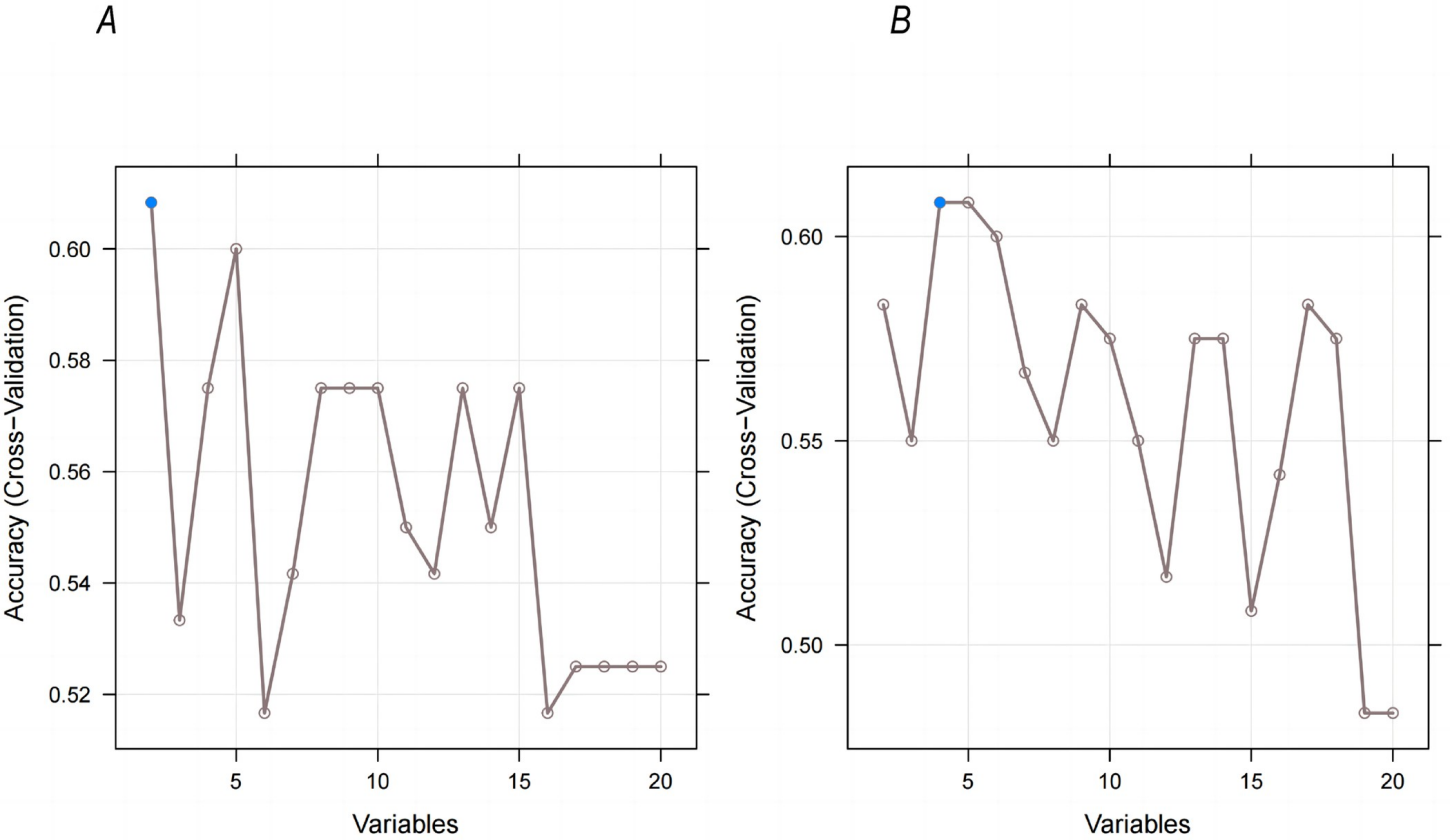
Screening of imaging features of the whole-tumor and whole-peritumor regions. Two imaging features (A) were screened in the whole tumor area, and four imaging features (B) were screened in the whole peritumoral area.

**Fig 5:**
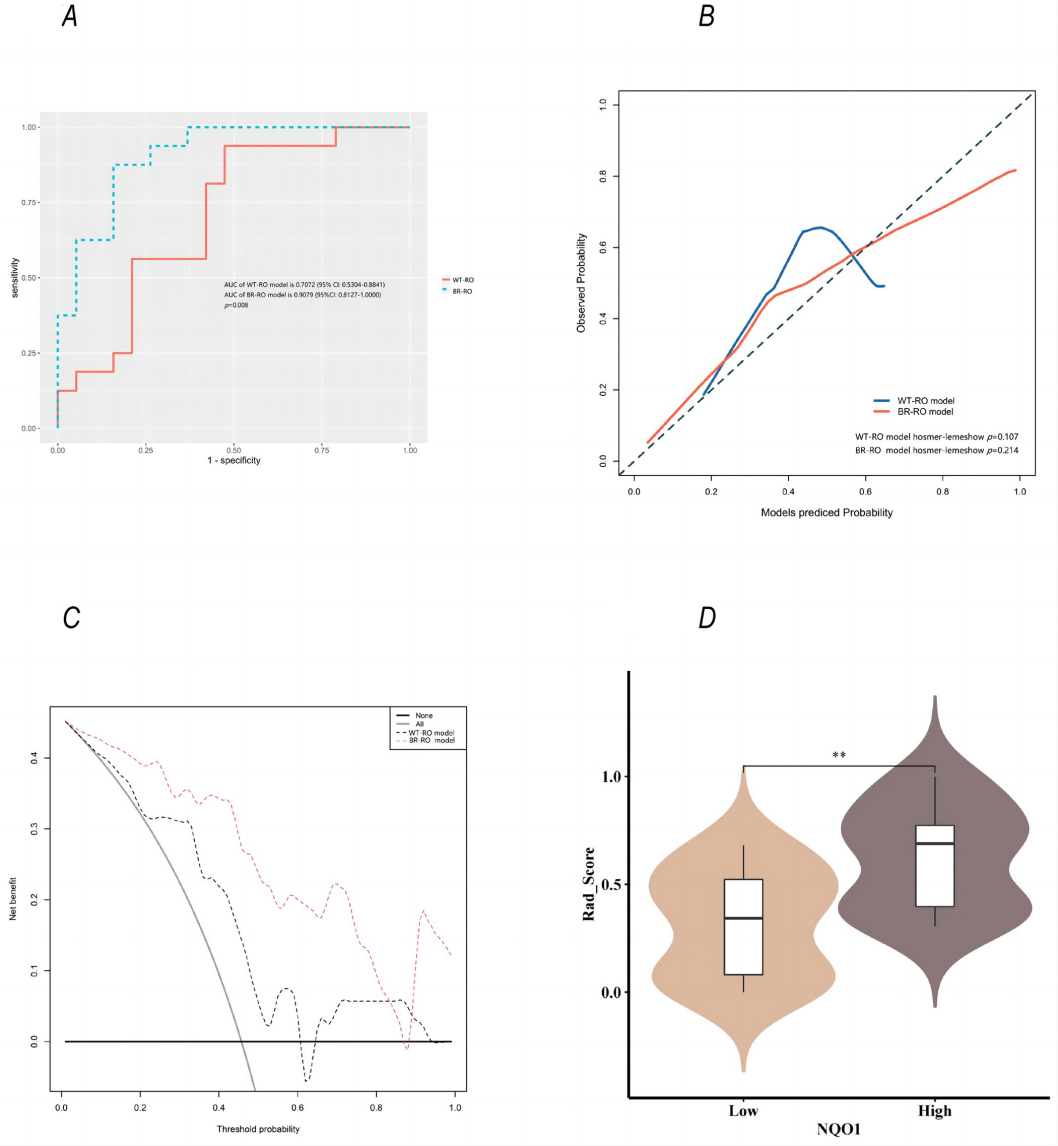
Comparison of WT-RO model and BR-RO model. (A) The AUC of the WT-RO model was 0.7072 (95% CI: 0.5304–0.8841), The AUC of BR-RO model was 0.9079 (95% CI 0.8127–1.0000). There was a significant difference in AUCs between the two models (*p* = 0.008). (B) The calibration curves and Hosmer–Lemeshow tests showed that the prediction probability of high expression of NQO1 by the radiomics prediction models was in good agreement with the true value (*p*>0.05). (C) Comparing the DCA curves of the WT-RO model and the BR-RO model, we found that the BR-RO model also seemed to be better than the WT-RO model. (D) The distribution of Rad_score was significantly different between high- and low-NQO1 groups (*p* = 0.002). The Rad score of the group with high expression of NQO1 was higher than that of the group with low expression of NQO1.

We established a prediction model called the whole-tumor regional radiomics (WT-RO) model using RFE with only two optimal features **(Fig 4A)**. The AUC of the WT-RO model was 0.7072 (95% CI: 0.5304–0.8841) **(Fig 5A)**. Based on the RFE analysis of whole-peritumor regional radiomic features **(Fig 4B)**, we constructed a bi-regional radiomics (BR-RO) model to predict NQO1 expression levels. This model included both the whole-tumor and whole-peritumor regions. There were six radiomic features: Whole_glszm_SmallAreaEmphasis, Whole_ngtdm_Coarseness, Whole_peri_glrlm_RunLengthNonUniformity, Whole_peri_ngtdm_Coarseness, Whole_peri_firstorder_TotalEnergy, and Whole_peri_glszm_SmallAreaLowGrayLevelEmphasis. The AUC of the BR-RO model was 0.9079 (95% CI: 0.8127–1.0000) **(Fig 5A)**. The accuracy, sensitivity, and specificity of the WT-RO model and the BR-RO model were 0.71, 0.94, and 0.53 and 0.86, 0.88, and 0.84 respectively, with thresholds of 0.332 and 0.404, respectively **(S1 Table)**.

### Evaluation of radiomic models

There was a significant difference in AUCs between the two models (*p* = 0.008) **(Fig 5A)**. The calibration curves and Hosmer–Lemeshow goodness-of-fit tests showed that the prediction probability of high-expression levels of NQO1 by the image omics prediction models was in good agreement with the true value (*p*>0.05) (**Fig 5B**). Comparing the DCA curves of the WT-RO and BR-RO models, the BR-RO model was better than the WT-RO model **(Fig 5C)**.

Owing to the better performance of the BR-RO model, the probability of predicting NQO1 expression levels was defined as the radiomics score. Radiomics score = −3.8126 × Whole_ngtdm_Coarseness + 1.5993 × Whole_glszm_SmallAreaEmphasis − 1.7211 × Whole_peri_glrlm_RunLengthNonUniformity + 0.4666 × Whole_peri_ngtdm_Coarseness − 0.2292 × Whole_peri_firstorder_TotalEnergy + 3.1423 × Whole_peri_glszm_SmallAreaLowGrayLevelEmphasis − 0.1927 **(Table 3)**.

**Table 3:** Selected features in radiomics model 2 and corresponding coefficients.

Radiomics features	Coefficient
Whole_ngtdm_Coarseness	-3.8126
Whole_glszm_SmallAreaEmphasis	1.5993
Whole_peri_glrlm_RunLengthNonUniformity	-1.7211
Whole_peri_ngtdm_Coarseness	0.4666
Whole_peri_firstorder_TotalEnergy	-0.2292
Whole_peri_glszm_SmallAreaLowGrayLevelEmphasis	3.1423

Next, we compared whether the radiomics score differed according to the expression of NQO1 between the two groups. The distribution of the score was significantly different between the two groups (*p* = 0.002) **(Fig 5D)**. The radiomic score of the high-NQO1 expression level group was higher.

**Fig 6:**
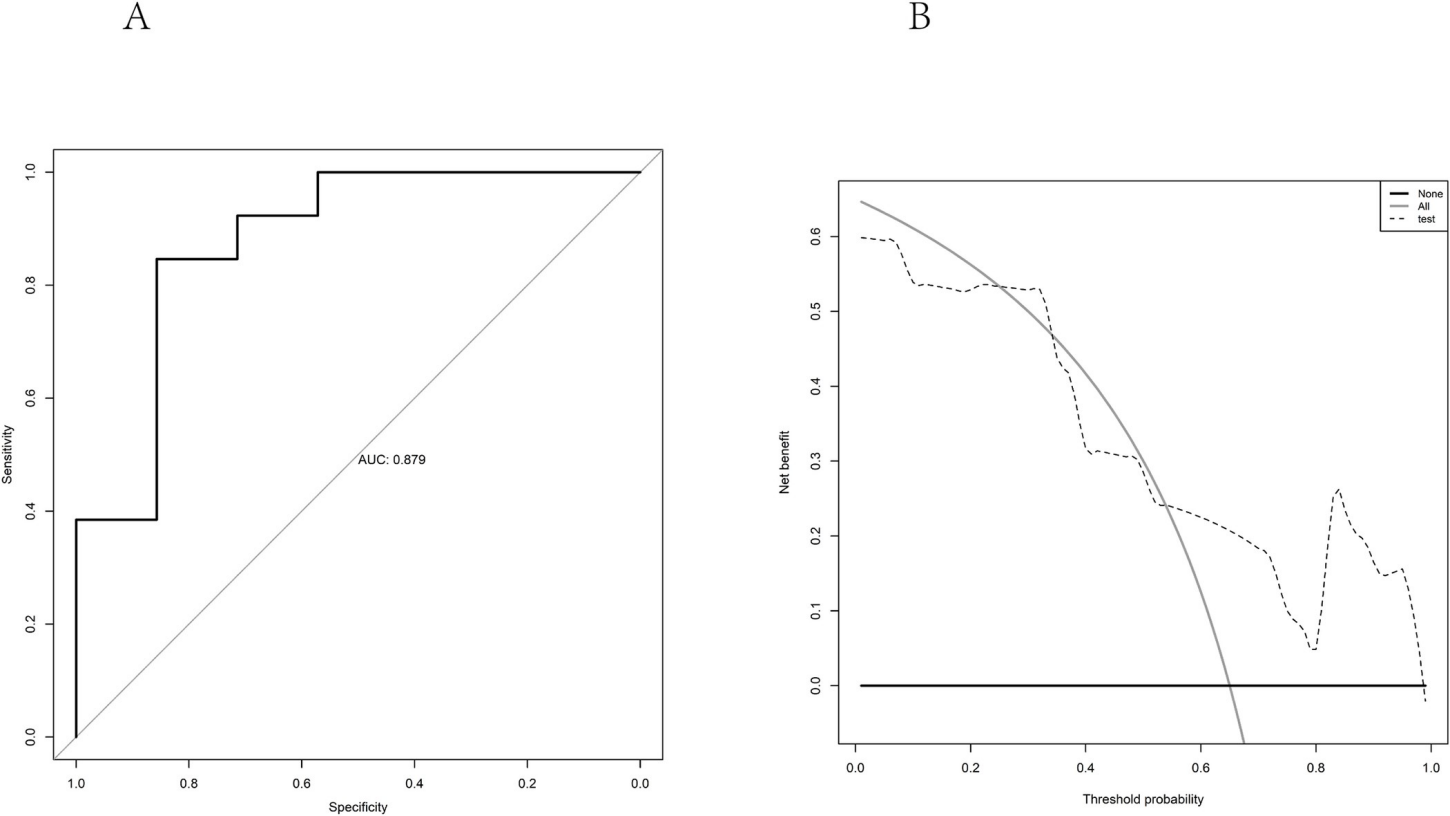
AUC and DCA curves for the external validation set. (A) The data verification of our center shows that this model has good predictive efficiency, with AUC is 0.879 (95% CI 0.698–1.000). (B) DCA curves of nomogram for predicting the probability of NQO1 level in validation set. The horizontal and vertical axes represent the threshold probability and net benefit, respectively. The lines between the horizontal axis and vertical axis display the benefit of different predicitive variables. the DCA curves show that if the threshold probability is 55%-99%, using this nomogram in the current study to predict high NQO1 express could add more benefit.

The data verification of our center showed that this model had good predictive efficiency, with an AUC of 0.8791 (95% CI 0.6979–1.0000) **(Fig 6A)**. The DCA curves showed that if the threshold probability is 55–99%, using this BR-RO model in the current study to predict high NQO1 expression can add more benefit **(Fig 6B)**.

## Discussion

The worldwide incidence of HCC is high, and it is estimated that more than 50% of HCC cases occur in China [2]. Studies have found that NQO1 plays an important role in many human cancers, including lung, breast, uterine cervical, and prostate cancer [22–25].To explore new prognostic markers to stratify patient prognosis in the present study, we used a noninvasive radiomic model to predict NQO1 expression levels and prognosis in HCC.

Bioinformatics analysis showed that the expression of NQO1 in HCC tumor tissues was higher than that in adjacent tissues and that this was an independent risk factor for poor prognosis of patients with HCC. This finding is consistent with those of previous research [6], which indicated that NQO1 may be an important target for liver cancer treatment in the future [26]. The patient characteristics, such as cancer grade, inflammation, residual tumor, and AFP level, did not significantly differ between the high- and low-NQO1-expression groups, except for vascular invasion. Furthermore, the correlation heat map showed that NQO1 was significantly correlated with vascular invasion. Overexpression of the NFE2L2 target gene *NQO1* in tumors was significantly associated with metastasis, vascular invasion, and poor outcome [27], which indicated the mechanism underlying the higher NQO1 level causing poor outcomes in patients with HCC. Further analysis of immune infiltration showed that the numbers of M0 macrophages and regulatory T cells were significantly increased in the group with high NQO1 expression, and the immune genes *TNFSF9* and *LGALS9* were significantly correlated with NQO1. Previous studies have shown that NQO1 plays an important role in specific immune cells, including macrophages, by suppressing Toll-like receptor-mediated innate immune responses [28], dendritic epidermal T cells [29], Th17 cells by regulating reactive oxygen species levels [30], and immune cells within the tumor microenvironment [31]. Combined with the results of the current study, it can be inferred that NQO1 may affect tumorigenesis and prognosis by influencing the function of immune cells.

Although various studies, including this one, have confirmed that NQO1 is closely related to HCC prognosis, noninvasive detection of NQO1 expression in liver tissues cannot be performed because of its dependence on surgical specimens. However, radiomics could be an excellent tool to solve this issue owing to its noninvasiveness, dynamic detection, and quantitative reflection of tumor characteristics. Medical images provide not only imaging data but also quantitative data upon conversion by certain technology. Analyzing such extracted data can reveal complex patterns that reflect biology at the macro and micro levels [32]. Radiomics is a high-throughput “image sequencing” technique that can obtain a large number of image parameters. In radiomic analysis, a statistical model is constructed to predict clinical events, such as prognosis or response. In clinical practice, radiomics technology is widely used to determine the prognosis of HCC [33, 34]. One prediction model was established using preoperative enhanced CT images and machine learning, which can accurately predict the preoperative pathological grade of liver cancer [35, 36]. A nomogram model integrating radiomics and clinical parameters has been developed to predict the survival of patients with combined hepatocellular-cholangiocarcinoma after hepatectomy [37]. Another radiomics model established using preoperative multiparameter magnetic resonance imaging can accurately predict the efficacy of unresectable HCC after transcatheter arterial chemoembolization [38].

Radiomics focuses on the noninvasive assessment of HCC genotype and immune profile, as well as treatment response prediction and assessment [33]. In the present study, preoperative arterial CT images of patients with HCC were used to delineate tumor areas and obtain whole-tumor imaging parameters. An RFE based on the mRMR algorithm was adopted. First, two parameters were obtained and incorporated into the logistic regression model; however, the predictive ability of this model was poor, with an AUC < 0.8. We propose that inclusion of peritumoral imaging parameters may improve the accuracy of this model. We obtained the whole-peritumor regional imaging parameters in the delineated tumor area, extending 3 mm outward. We created a BR-RO model to predict NQO1 expression levels, which included both the whole-tumor and whole-peritumor regions. Finally, six parameters from preoperative arterial CT images (Whole_glszm_SmallAreaEmphasis, Whole_ngtdm_Coarseness, Whole_peri_glrlm_RunLengthNonUniformity, Whole_peri_ngtdm_Coarseness, Whole_peri_firstorder_TotalEnergy, and Whole_peri_glszm_SmallAreaLowGrayLevelEmphasis) were included to establish a bi-regional model that could accurately predict the higher expression levels of NQO1 in patients with HCC, with an AUC of 0.9079. Furthermore, the fine calibration curves and DCA curves confirm the excellent performance of the new bi-regional model, which can predict higher expression levels of NQO1 noninvasively in patients with HCC. Compared to the previous studies [39, 40], our results suggested that heterogeneity does exist in peritumoral tissue, indicating that the radiomic features of the bi-regional area from CT images can be utilized to predict the NQO1 expression. Similar findings have been found in other tumors [41–43].We believe that this novel model can be used as a good strategy to noninvasively judge HCC prognosis and provide more accurate decision-making information for HCC treatment.

Although the radiomic model performed well, this study had several limitations. First, clinical data, including CT images, were acquired from public datasets, which inevitably contain large variances in image quality, which may impact the predictive model. Second, our study only included arterial phase images and lacked plain and venous CT images. We plan to include all CT images in future studies. Finally, although external data verified that the model had good predictive efficiency further multicenter studies are required to optimize this model.

Artificial intelligence (AI) aims to use computers to simulate human learning activities by identifying existing knowledge, acquiring new knowledge, constantly improving performance, and achieving self-improvement. AI uses its powerful data analysis and learning capabilities to mine and analyze massive high-latitude medical data to form various prediction models [44–46]. In future, we plan to include more imaging data of dynamic CT and implement AI technology to expect its ability to predict NQO1.

In conclusion, the present study revealed a significant correlation between CT images and the expression level of NQO1, which could indirectly reflect the prognosis of patients with HCC. The predictive model based on arterial phase CT imaging features has good stability and diagnostic efficiency and is a potential means to noninvasively identify the expression levels of NQO1 in liver cancer tissues.

## Supporting information

S1 Table The predictive performance/efficacy of each model. (DOCX)

S1 Data (RAR)

## References

[1] Torre LA, Bray F, Siegel RL, Ferlay J, Lortet-Tieulent J, Jemal A. Global cancer statistics, 2012. CA Cancer J Clin. 2015. 65(2): 87–108. doi: 10.3322/caac.21262 25651787

[2] Bray F, Ferlay J, Soerjomataram I, Siegel RL, Torre LA, Jemal A. Global cancer statistics 2018: GLOBOCAN estimates of incidence and mortality worldwide for 36 cancers in 185 countries. CA Cancer J Clin. 2018. 68(6): 394–424. doi: 10.3322/caac.21492 30207593

[3] Sung H, Ferlay J, Siegel RL, et al. Global Cancer Statistics 2020: GLOBOCAN Estimates of Incidence and Mortality Worldwide for 36 Cancers in 185 Countries. CA Cancer J Clin. 2021. 71(3): 209–249. 33538338 10.3322/caac.21660

[4] Jaiswal AK, McBride OW, Adesnik M, Nebert DW. Human dioxin-inducible cytosolic NAD(P)H:menadione oxidoreductase. cDNA sequence and localization of gene to chromosome 16. J Biol Chem. 1988. 263(27): 13572–13578. 2843525

[5] Fagerberg L, Hallström BM, Oksvold P, et al. Analysis of the human tissue-specific expression by genome-wide integration of transcriptomics and antibody-based proteomics. Mol Cell Proteomics. 2014. 13(2): 397–406. 24309898 10.1074/mcp.M113.035600 PMC3916642

[6] Lin L, Sun J, Tan Y, et al. Prognostic implication of NQO1 overexpression in hepatocellular carcinoma. Hum Pathol. 2017. 69: 31–37. doi: 10.1016/j.humpath.2017.09.002 28964792

[7] Dimri M, Humphries A, Laknaur A, et al. NAD(P)H Quinone Dehydrogenase 1 Ablation Inhibits Activation of the Phosphoinositide 3-Kinase/Akt Serine/Threonine Kinase and Mitogen-Activated Protein Kinase/Extracellular Signal-Regulated Kinase Pathways and Blocks Metabolic Adaptation in Hepatocellular Carcinoma. Hepatology. 2020. 71(2): 549–568. doi: 10.1002/hep.30818 31215069 PMC6920612

[8] Li WY, Zhou HZ, Chen Y, et al. NAD(P)H: Quinone oxidoreductase 1 overexpression in hepatocellular carcinoma potentiates apoptosis evasion through regulating stabilization of X-linked inhibitor of apoptosis protein. Cancer Lett. 2019. 451: 156–167. doi: 10.1016/j.canlet.2019.02.053 30867140

[9] Dou L, Liu H, Wang K, et al. Albumin binding revitalizes NQO1 bioactivatable drugs as novel therapeutics for pancreatic cancer. J Control Release. 2022. 349: 876–889. doi: 10.1016/j.jconrel.2022.07.033 35907592

[10] Jiao B, Liu K, Gong H, et al. Bladder cancer selective chemotherapy with potent NQO1 substrate co-loaded prodrug nanoparticles. J Control Release. 2022. 347: 632–648. doi: 10.1016/j.jconrel.2022.05.031 35618186

[11] Yang X, Duan J, Wu L. Research advances in NQO1-responsive prodrugs and nanocarriers for cancer treatment. Future Med Chem. 2022. 14(5): 363–383. doi: 10.4155/fmc-2021-0289 35102756

[12] Zhu H, Lu L, Zhu W, et al. Design and synthesis of NAD(P)H: Quinone oxidoreductase (NQO1)-activated prodrugs of 23-hydroxybetulinic acid with enhanced antitumor properties. Eur J Med Chem. 2022. 240: 114575. doi: 10.1016/j.ejmech.2022.114575 35803175

[13] Kim S, Shin J, Kim DY, Choi GH, Kim MJ, Choi JY. Radiomics on Gadoxetic Acid-Enhanced Magnetic Resonance Imaging for Prediction of Postoperative Early and Late Recurrence of Single Hepatocellular Carcinoma. Clin Cancer Res. 2019. 25(13): 3847–3855. doi: 10.1158/1078-0432.CCR-18-2861 30808773

[14] Gao L, Xiong M, Chen X, et al. Multi-Region Radiomic Analysis Based on Multi-Sequence MRI Can Preoperatively Predict Microvascular Invasion in Hepatocellular Carcinoma. Front Oncol. 2022. 12: 818681. doi: 10.3389/fonc.2022.818681 35574328 PMC9094629

[15] Meng XP, Tang TY, Ding ZM, et al. Preoperative Microvascular Invasion Prediction to Assist in Surgical Plan for Single Hepatocellular Carcinoma: Better Together with Radiomics. Ann Surg Oncol. 2022. 29(5): 2960–2970. doi: 10.1245/s10434-022-11346-1 35102453

[16] Khemlina G, Ikeda S, Kurzrock R. The biology of Hepatocellular carcinoma: implications for genomic and immune therapies. Mol Cancer. 2017. 16(1): 149. doi: 10.1186/s12943-017-0712-x 28854942 PMC5577674

[17] Kurebayashi Y, Ojima H, Tsujikawa H, et al. Landscape of immune microenvironment in hepatocellular carcinoma and its additional impact on histological and molecular classification. Hepatology. 2018. 68(3): 1025–1041. doi: 10.1002/hep.29904 29603348

[18] Vivian J, Rao AA, Nothaft FA, et al. Toil enables reproducible, open source, big biomedical data analyses. Nat Biotechnol. 2017. 35(4): 314–316. doi: 10.1038/nbt.3772 28398314 PMC5546205

[19] Clark K, Vendt B, Smith K, et al. The Cancer Imaging Archive (TCIA): maintaining and operating a public information repository. J Digit Imaging. 2013. 26(6): 1045–1057. doi: 10.1007/s10278-013-9622-7 23884657 PMC3824915

[20] Li Q, Luo G, Li J. Evaluation of Therapeutic Effects of Computed Tomography Imaging Classification Algorithm-Based Transcatheter Arterial Chemoembolization on Primary Hepatocellular Carcinoma. Comput Intell Neurosci. 2022. 2022: 5639820. doi: 10.1155/2022/5639820 35498180 PMC9054411

[21] DeLong ER, DeLong DM, Clarke-Pearson DL. Comparing the areas under two or more correlated receiver operating characteristic curves: a nonparametric approach. Biometrics. 1988. 44(3): 837–845. 3203132

[22] Ma Y, Kong J, Yan G, et al. NQO1 overexpression is associated with poor prognosis in squamous cell carcinoma of the uterine cervix. BMC Cancer. 2014. 14(1): 414. doi: 10.1186/1471-2407-14-414 24912939 PMC4058702

[23] Thapa D, Meng P, Bedolla RG, Reddick RL, Kumar AP, Ghosh R. NQO1 suppresses NF-κB-p300 interaction to regulate inflammatory mediators associated with prostate tumorigenesis. Cancer Res. 2014. 74(19): 5644–5655. 25125658 10.1158/0008-5472.CAN-14-0562 PMC4184940

[24] Yang Y, Zhang Y, Wu Q, et al. Clinical implications of high NQO1 expression in breast cancers. J Exp Clin Cancer Res. 2014. 33(1): 14. doi: 10.1186/1756-9966-33-14 24499631 PMC3944477

[25] Li Z, Zhang Y, Jin T, et al. NQO1 protein expression predicts poor prognosis of non-small cell lung cancers. BMC Cancer. 2015. 15: 207. doi: 10.1186/s12885-015-1227-8 25880877 PMC4396547

[26] Wang X, Liu Y, Han A, et al. The NQO1/p53/SREBP1 axis promotes hepatocellular carcinoma progression and metastasis by regulating Snail stability. Oncogene. 2022. 41(47): 5107–5120. doi: 10.1038/s41388-022-02477-6 36253445

[27] Eichenmüller M, Trippel F, Kreuder M, et al. The genomic landscape of hepatoblastoma and their progenies with HCC-like features. J Hepatol. 2014. 61(6): 1312–1320. doi: 10.1016/j.jhep.2014.08.009 25135868

[28] Kimura A, Kitajima M, Nishida K, et al. NQO1 inhibits the TLR-dependent production of selective cytokines by promoting IκB-ζ degradation. J Exp Med. 2018. 215(8): 2197–2209. 29934320 10.1084/jem.20172024 PMC6080903

[29] Kitajima M, Kimura A, Suzuki H. Cutting Edge: Nqo1 Regulates Irritant Contact Hypersensitivity against Croton Oil through Maintenance of Dendritic Epidermal T Cells. J Immunol. 2018. 200(5): 1555–1559. doi: 10.4049/jimmunol.1701389 29378915

[30] Nishida-Tamehiro K, Kimura A, Tsubata T, Takahashi S, Suzuki H. Antioxidative enzyme NAD(P)H quinone oxidoreductase 1 (NQO1) modulates the differentiation of Th17 cells by regulating ROS levels. PLoS One. 2022. 17(7): e0272090. doi: 10.1371/journal.pone.0272090 35905076 PMC9337673

[31] Kaghazchi B, Um IH, Elshani M, Read OJ, Harrison DJ. Spatial Analysis of NQO1 in Non-Small Cell Lung Cancer Shows Its Expression Is Independent of NRF1 and NRF2 in the Tumor Microenvironment. Biomolecules. 2022. 12(11): 1652. doi: 10.3390/biom12111652 36359002 PMC9687417

[32] Gillies RJ, Kinahan PE, Hricak H. Radiomics: Images Are More than Pictures, They Are Data. Radiology. 2016. 278(2): 563–577. doi: 10.1148/radiol.2015151169 26579733 PMC4734157

[33] Borhani AA, Catania R, Velichko YS, Hectors S, Taouli B, Lewis S. Radiomics of hepatocellular carcinoma: promising roles in patient selection, prediction, and assessment of treatment response. Abdom Radiol (NY). 2021. 46(8): 3674–3685. doi: 10.1007/s00261-021-03085-w 33891149

[34] Lewis S, Hectors S, Taouli B. Radiomics of hepatocellular carcinoma. Abdom Radiol (NY). 2021. 46(1): 111–123. doi: 10.1007/s00261-019-02378-5 31925492

[35] Mao B, Zhang L, Ning P, et al. Preoperative prediction for pathological grade of hepatocellular carcinoma via machine learning-based radiomics. Eur Radiol. 2020. 30(12): 6924–6932. doi: 10.1007/s00330-020-07056-5 32696256

[36] Wei J, Ji Q, Gao Y, et al. A multi-scale, multi-region and attention mechanism-based deep learning framework for prediction of grading in hepatocellular carcinoma. Med Phys. 2022. 50(4): 2290–2302 doi: 10.1002/mp.16127 36453607

[37] Tang YY, Zhao YN, Zhang T, Chen ZY, Ma XL. Comprehensive radiomics nomogram for predicting survival of patients with combined hepatocellular carcinoma and cholangiocarcinoma. World J Gastroenterol. 2021. 27(41): 7173–7189. doi: 10.3748/wjg.v27.i41.7173 34887636 PMC8613648

[38] Sun Y, Bai H, Xia W, et al. Predicting the Outcome of Transcatheter Arterial Embolization Therapy for Unresectable Hepatocellular Carcinoma Based on Radiomics of Preoperative Multiparameter MRI. J Magn Reson Imaging. 2020. 52(4): 1083–1090. doi: 10.1002/jmri.27143 32233054

[39] Tong X, Li J. Noninvasively predict the micro-vascular invasion and histopathological grade of hepatocellular carcinoma with CT-derived radiomics. Eur J Radiol Open 2022; 9: 100424. doi: 10.1016/j.ejro.2022.100424 35600083 PMC9120240

[40] Feng Z, Li H, Liu Q, et al. CT Radiomics to Predict Macrotrabecular-Massive Subtype and Immune Status in Hepatocellular Carcinoma. Radiology 2023; 307(1): e221291. doi: 10.1148/radiol.221291 36511807

[41] Zhou Z, Qian X, Hu J, et al. CT-based peritumoral radiomics signatures for malignancy grading of clear cell renal cell carcinoma. Abdom Radiol (NY) 2021; 46(6): 2690–2698. doi: 10.1007/s00261-020-02890-z 33427908

[42] Long H, Zhang P, Bi Y, et al. MRI radiomic features of peritumoral edema may predict the recurrence sites of glioblastoma multiforme. Front Oncol 2022; 12: 1042498. doi: 10.3389/fonc.2022.1042498 36686829 PMC9845721

[43] Le N, Hung T, Do DT, et al. Radiomics-based machine learning model for efficiently classifying transcriptome subtypes in glioblastoma patients from MRI. Comput Biol Med 2021; 132: 104320. doi: 10.1016/j.compbiomed.2021.104320 33735760

[44] Zhang Z, Wei X. Artificial intelligence-assisted selection and efficacy prediction of antineoplastic strategies for precision cancer therapy. Semin Cancer Biol 2023; 90: 57–72. doi: 10.1016/j.semcancer.2023.02.005 36796530

[45] Shao J, Ma J, Zhang Q, et al. Predicting gene mutation status via artificial intelligence technologies based on multimodal integration (MMI) to advance precision oncology. Seminars in cancer biology 2023;91: 1–15 doi: 10.1016/j.semcancer.2023.02.006 36801447

[46] Calderaro J, Seraphin TP, Luedde T, et al. Artificial intelligence for the prevention and clinical management of hepatocellular carcinoma. Journal of hepatology 2022; 76(6): 1348–1361 doi: 10.1016/j.jhep.2022.01.014 35589255 PMC9126418

