# Supplementary material for: CT radiomics for noninvasively predicting NQO1 expression levels in hepatocellular carcinoma
Source: PLoS One. 2023 Sep 11;18(9):e0290900. doi: 10.1371/journal.pone.0290900 (PMC10495018; doi:10.1371/journal.pone.0290900)
Supplement: S1 Table — (DOCX) [file pone.0290900.s001.docx]

| Supplementary table: the predictive performance/efficacy of each model | | | | | |
| --- | --- | --- | --- | --- | --- |
| Radiomics model | AUC | Accuracy | Sensitivity | Specificity | Threshold |
| BR-RO model | 0.908 (95% CI: 0.813–1.000) | 0.86 | 0.88 | 0.84 | 0.404 |
| WT-RO model | 0.707 (95% CI: 0.530–0.884) | 0.71 | 0.94 | 0.53 | 0.332 |
